# Strengthening and Toughening of Polylactide/Sisal Fiber Biocomposites via in-situ Reaction with Epoxy-Functionalized Oligomer and Poly (butylene-adipate-terephthalate)

**DOI:** 10.3390/polym11111747

**Published:** 2019-10-24

**Authors:** Hongwu Wu, Mingyang Hao

**Affiliations:** 1 The Key Laboratory of Polymer Processing Engineering of the Ministry of Education, South China University of Technology, Guangzhou 510640, China; 2 National Engineering Research Center of Novel Equipment for Polymer Processing, South China University of Technology, Guangzhou 510640, China; 3 Guangdong Provincial Key Laboratory of Technique and Equipment for Macromolecular Advanced Manufacturing, South China University of Technology, Guangzhou 510640, China

**Keywords:** polymer-matrix composites (PMCs), fiber/matrix bond, microstructures, interface/interphase, mechanical properties

## Abstract

With the addition of poly (butylene-adipate-terephthalate) (PBAT) and a commercial grade epoxy-functionalized oligomer Joncryl ADR^@^-4368 (ADR), a blend of polylactic acid (PLA) and sisal fibers (SF) were melt-prepared via in-situ reactive process to improve the toughness and interfacial bonding of polylactide/sisal fiber composites. Fourier Transform infrared spectroscopy (FTIR) analysis demonstrated chemical bonding between sisal fibers and matrix, and scanning electron microscope characterization indicated the enhancement of interfacial adhesion between PLA matrix and sisal fibers. The micro-debonding test proved that the interfacial adhesion between PLA and SF was improved because of ADR. The presence of ADR behaved like a hinge among sisal fibers and matrix via an in-situ interfacial reaction, and compatibility between PLA and PBAT was also augmented. The introduction of PBAT exerted a plasticization effect on composites. Therefore, the toughness of PLA/SF composites was significantly elevated, while the tensile strength of composites could be well preserved. The paper focused on the demonstration of interfacial interaction and structure–properties relationship of the composites.

## 1. Introduction

The interfacial adhesion inside composites, together with the intrinsic properties of components have an essential effect on the mechanical properties of fiber-reinforced polymer composites [1,2]. When a load is applied onto a fiber-reinforced polymer composites sample, stress will be transferred from polymer matrix to fibers through the interfaces. Good interfacial adhesion between the polymer matrix and fibers will offer an efficient stress transference from matrix to fibers whereby the mechanical performance of composites increases, which has been proven by numerous [3]. The hydrophilic nature of plant fiber and the hydrophobic nature of polymer matrix generally result in poor interfacial compatibility between plant fiber and polymer matrix in plant fiber-reinforced polymer composites. Inside plant fiber-reinforced polymer composites, fibers and polymer are physical and/or chemically combined, and the interface can be considered as a diffusion or reaction zone. The poor interface is widely regarded as the most important mechanisms of bond failure. 

Many pretreatment methods have been applied to plant fibers prior to the blending of plant fibers and polymer matrix in order to improve the interfacial interaction of plant fiber-reinforced polymer composites [4]. Chemical treatment of plant fiber surface has been regarded as an effective modus operandi to improve interfacial compatibility and adhesion of plant fiber-reinforced polymer composites [5]. Chemical pretreatment methods that were developed for plant fiber include silane treatment [6,7], acetylation treatment [8], benzoylation treatment [9], acrylation and acrylonitrile grafting [10,11], dopamine treatment [12], N-methylol acrylamide grafting [13], etc. Alkali treatment using sodium hydroxide can remove hemicellulose, lignin, waxes, and impurities from plant fiber surfaces. Therefore, it will improve the surface roughness of plant fibers and expose more reactive functional groups on the fiber surface. Nowadays, alkali treatment has been broadly utilized as an essential procedure for the preparation of plant fibers/polymer composites [14,15,16]. Chemical pretreatment could augment the interfacial adhesion of plant fibers and polymer matrices, but it usually weakens the fiber strength itself simultaneously. Furthermore, chemical pretreatments are commonly inefficient, because they generally necessitate a relative long time of soaking plant fibers in corresponding solution. In addition, the organic solvents that were used in chemical pretreatment are usually not environmentally benign. However, an in-situ reactive compatibilization can enhance the interfacial properties of composites without the disadvantages of chemical pretreatment during processing, and thus constitutes a promising method for preparing plant fiber-reinforced polymer composites.

In this study, polylactic acid and sisal fibers were utilized to fabricate bio-based plant fiber reinforced polymer composites. Plant fiber-reinforced polylactic acid composite is a kind of green and fully biodegradable material. However, the low toughness of plant fiber-reinforced polylactic acid (PLA) presents a disadvantage for application due to the inherent brittleness and low toughness of PLA. Polylactic acid resin and sisal fibers were melt-blended via in-situ reactive interfacial compatibilization with the addition of ADR oligomer and poly (butylene-adipate-terephthalate) (PBAT) resin to improve the interfacial adhesion and the toughness of PLA/sisal fiber composites. Joncryl ADR^®^-4368 is a kind of commercial grade multi-epoxy-functionalized styrene-acrylic oligomer (ADR) [17,18,19]. We consider that ADR has the potential to be an efficient reactive compatibilizer in plant fiber reinforced polymer composites. The in-situ reaction processing method offers the possibility of augmenting the interfacial compatibility of sisal fibers with PLA/PBAT matrix via interfacial reaction between reactive additive and plant fiber, and between additive and PLA/PBAT. The experiment was also designed to realize the reactive toughening of composites at the same time. The phase morphology and mechanical properties of the composites were analyzed. The paper focused on the demonstration of interfacial interaction and structure–properties relationship of the composites.

## 2. Experimental Section

### 2.1. Materials

A semi-crystalline extrusion grade of PLA (trade name 4032D) with 1.2–1.6% D-isomer lactide and density of 1.25 g/cm^3^, from Nature Works LLC (Minnetonka, MN, U.S.A.), was used. It was air-dried in an oven at 80 °C for 8 h prior to use. Poly (butylene-adipate-terephthalate) (PBAT, *T_g_* = −29 °C, Ecoflex FBX 7011) produced by BASF (Ludwigshafen, Germany) was purchased in the market. Sisal fibers were purchased from Dongfang Sisal Co. (Guangdong, China), and the properties of the fiber provided by the supplier are shown in Table 1. Joncryl ADR^®^-4368 was supplied by Shanghai Kingpont Chemical Co. (Shanghai, China).

### 2.2. Preparation of the Composites

The sisal fibers were cut short in a length of 6 mm and then soaked in sodium hydroxide solution (5 wt%) for 1 h to remove lignin, pectin and waxy substances on the surface. After that, they were vacuum-dried at 80 °C for 8 h. Melt blending of PLA, PBAT, sisal fibers (SF), and ADR was performed while using an internal mixer (Poton 100, POTOP Experimental Analysis Instrument Co., Ltd, Guangzhou, China) at 200 °C. for 5 min. with a roller speed of 80 rpm. A series of PLA/SF, PLA/SF/ADR, and PLA/PBAT/SF/ADR composites were prepared with different PBAT addition, different SF content, and a constant ADR ratio of 0.6 wt% referred to the total weight of PLA, PBAT, and SF. The dosage of PBAT is from 0 to 20 wt% and the SF content is from 10 to 40 wt%, respectively. The obtained mixtures were compression-molded at 200 °C for 3 min. under 10 MPa into standard specimens for mechanical tests. Five specimens were tested for each composite.

### 2.3. Morphological Characterization

The morphologies of impact and tensile fracture surface of the specimens were observed and recorded with a scanning electron microscope (SEM, FEI Quatan 250, FEI Company, Hillsboro, OR, USA.) after mechanical tests. Fracture surfaces were sputtered with gold to provide enhanced conductivity prior to SEM observation.

### 2.4. Fourier Transform Infrared Spectroscopy (FTIR) Measurement

Using dichloromethane as a solvent, sisal fibers were extracted from composites by Soxhlet extraction to evaluate the interfacial bonding between the sisal fibers and PLA/PBAT. The obtained sisal fibers were then dried in a vacuum oven at 80 °C for 8 h and ground into powder for FTIR analysis. Finally, they were characterized with a FTIR spectroscope (Nexus 670, Thermo Nicolet Co. Ltd., Madison, WI, USA; KBr powder) over a range of 4000–400 cm^−1^. FTIR characterization of PLA resin was also performed for comparison.

### 2.5. Micro-Debonding Test

Micro-debonding test was performed to verify the enhancement of interfacial shearing strength between SF and PLA with the addition of ADR. At first, ADR was ground into powder and put in a bag. Sisal microfibers were inserted into the bag to be covered with ADR. PLA was placed in an oven at 190 °C for 10 min. to be melted. Some filaments were drawn out from the molten PLA with tweezers and then bound onto the ADR-covered fibers. The samples without ADR were prepared as the control. The above samples were placed in a vacuum oven at 200 °C for 5 min. PLA melted into droplets and attached onto the surface of sisal fibers. The length of the droplets along fibers was from 0.83mm to 1.25mm. Finally, the micro-debonding test was carried out on a universal material machine (Instron 5566, INSTRON Corporation, Boston, MA, USA). The sample was clamped by a specific jig with a pore that a single fiber could pass through freely, but not the droplets. Therefore, the sisal fibers would be pulled out of the droplets when enough tension was applied at one end of the sisal fibers. The tensile rate was set to 0.5 mm/min. The interfacial shear strength (IFSS) between SF and PLA could be calculated by Equation 1.

(1) $$IFSS=\frac{F_{MAX}}{L_{e}\cdot d_{f}\cdot \pi }$$

where *F_MAX_* is the maximum load, *L_e_* is the length of the droplet that embeds the fiber, and *d_f_* is the diameter of the fiber. *L_e_* and *d_f_* were measured by stereomicroscope (OLYMPUS SZ61, OLYMPUS Corporation, Tokyo, Japan)

Five samples with ADR and another five samples without ADR were tested. Then, average values were calculated and standard errors were obtained.

### 2.6. Measurements of Mechanical Properties

Notched Izod impact tests were carried out following ISO 180, while using a 5.5 J pendulum at room temperature. The tensile tests and flexural tests were performed on the Instron 5566 universal tensile testing machine according to ISO 527-2 with a crosshead speed of 2 mm/min. and ISO 14125 with a speed of 2 mm/min. At least five specimens for each composite were tested. Average values were calculated and standard errors were obtained.

## 3. Results and Discussion

### 3.1. Morphology Analysis

**Figure 1:**
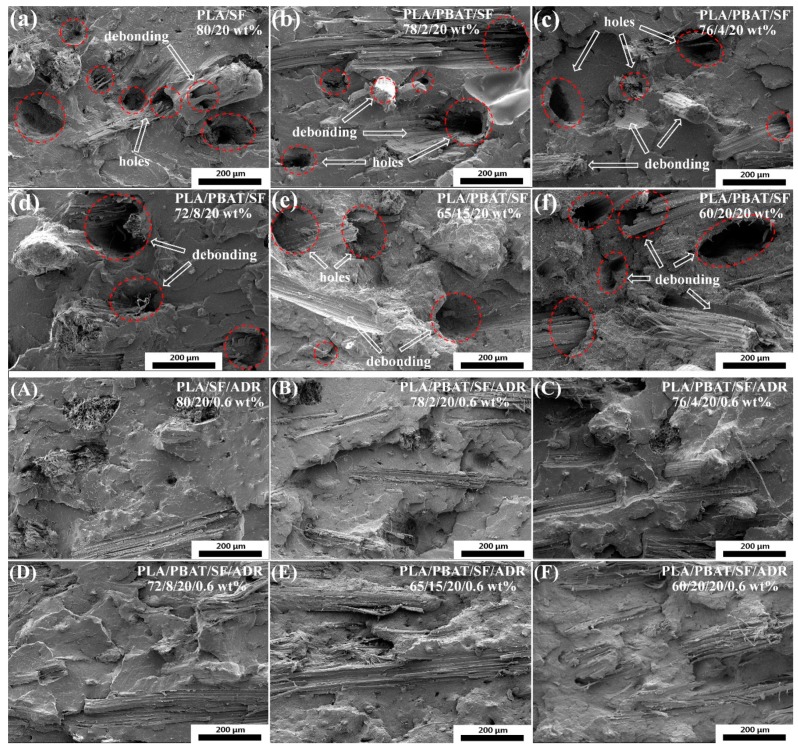
Impact fracture surfaces of polylactic acid/sisal fibers (PLA/SF) composites (**a**); PLA/poly (butylene-adipate-terephthalate)/SF (PLA/PBAT/SF) composites (**b**–**f**); PLA/SF/commercial grade multi-epoxy-functionalized styrene-acrylic oligomer (PLA/SF/ADR) composites (**A**); PLA/PBAT/SF/ADR composites (**B**–**F**). 500× magnification.

Figure 1a–f show the impact fracture surfaces of the PLA/SF composites and PLA/PBAT/SF composites, with 500× magnification. For PLA/SF composites, many fibers were directly pulled out of the matrix on the fracture surfaces, and numerous holes and debonding were formed, as can be seen in Figure 1a. These phenomena reflected the poor interfacial adhesion between PLA matrix and sisal fibers. For PLA/PBAT/SF composites (Figure 1b–f), the debonding of sisal fibers and matrix also occurred on the fracture surface, and the incorporation of PBAT did not improve the interfacial adhesion of composites. As a comparison, Figure 1A–F present the impact fracture surfaces of PLA/SF/ADR composites and PLA/PBAT/SF/ADR composites. It can be seen from Figure 1A–F that the fibers’ pull-out phenomena dramatically decreased. The fibers were tightly connected with the matrix or underlain the matrix, and they tended to be broken and torn up in the composites. The fracture surfaces became more uneven and PLA showed a feature of ductile fracture. These results demonstrated that the addition of ADR oligomer augmented the interfacial adhesion of PLA/SF/ADR and PLA/PBAT/SF/ADR composites.

**Figure 2:**
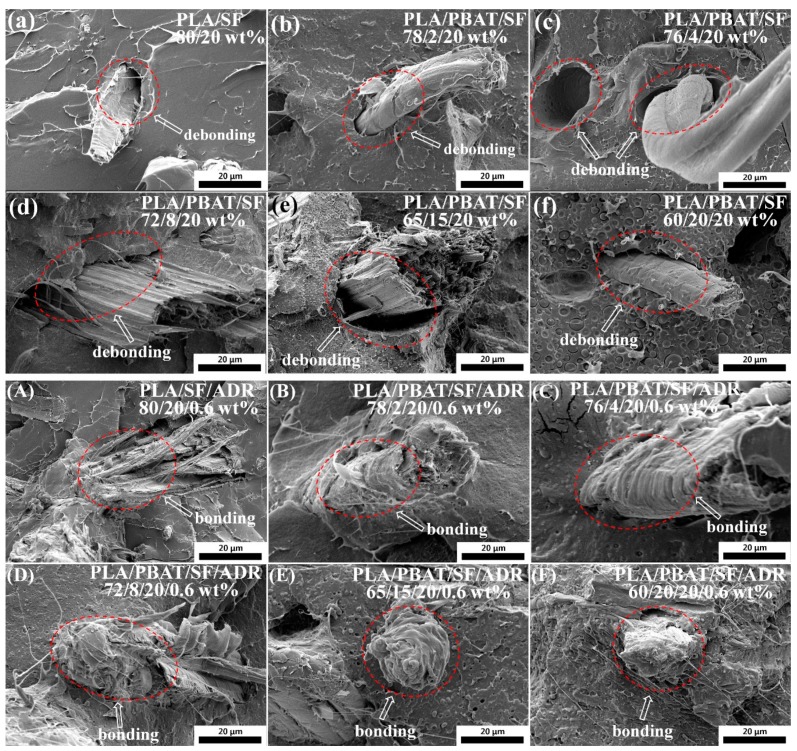
Fiber morphology on impact fracture surfaces PLA/SF composites (**a**); PLA/PBAT/SF composites (**b**–**f**); PLA/SF/ADR composites (**A**); PLA/PBAT/SF/ADR composites (**B**–**F**). 5000× magnification.

Figure 2 shows the fiber morphology on the impact fracture surfaces with 5000× magnification. The debonding of sisal fibers and matrix can be observed in PLA/SF and PLA/PBAT/SF composites, and the fibers on the fracture surfaces nearly retain the original morphology (Figure 2a–f). However, the fibers and matrix were well bonded in interphase for PLA/SF/ADR and PLA/PBAT/SF/ADR composites (Figure 2A–F). Furthermore, it was found that part of the polymer matrix tended to weld on the fiber surface in PLA/PBAT/SF/ADR composites (Figure 2B–F), and the adhered resin exhibited elasto-plastic deformation, which indicated the plasticization of PLA matrix via the addition of PBAT. It can also be seen that the impact fracture surface tended to be rougher as PBAT content increased, obviously presenting a typical feature of ductile fracture. These phenomena also represented the plasticization effect of PBAT resin on PLA matrix with the use of ADR. Wu et al. found that a unique self-weld fiber structure could be observed when PA6 was incorporated into Polystyrene (PS)/GF composite as PA6 weld GF into a continuous network structure [20]. Fu et al. also found that poly(ether)urethane could play a role of “solder” to weld the carbon fibers into a kind of self-welded fiber structure during their investigation of incorporating poly(ether)urethane into polylactide/carbon fiber composites [21]. Currently, the SEM images (Figure 2B–F) displayed that a similar morphology was obtained in this study for PLA/PBAT/SF/ADR composites via in-situ reaction processing. Part of PBAT concentrated on the sisal fiber surface and welded the sisal fibers to the PLA matrix to form a self-welded fiber structure with the employment of ADR, which improved the interfacial interaction between fibers and polymer matrix, as well as between PLA and PBAT. The self-welded fiber structure would contribute to the enhancement of tensile strength and toughness of composites.

**Figure 3:**
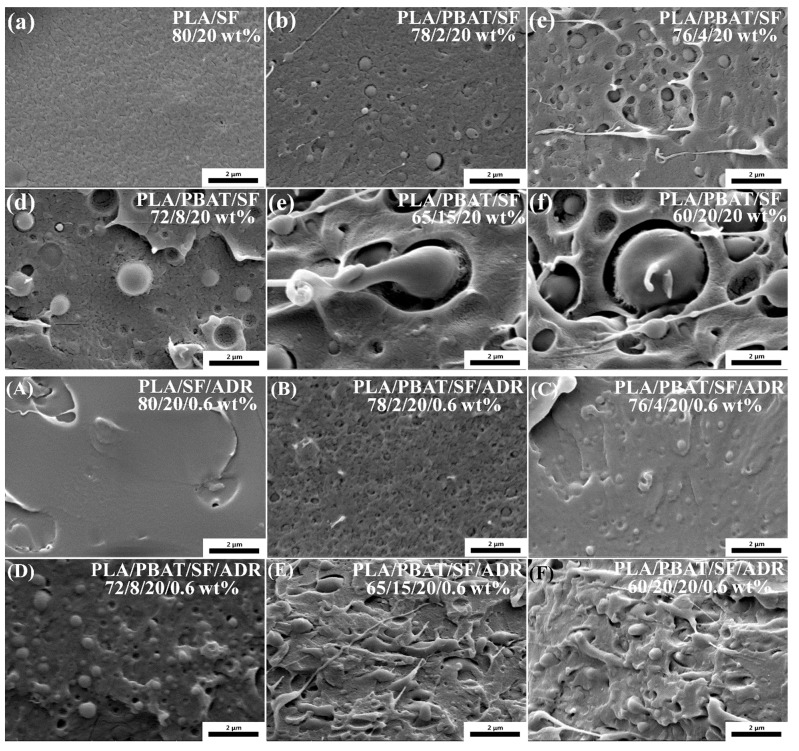
High magnification scanning electron microscope (SEM) micrographs of composites: PLA/SF composites (**a**); PLA/PBAT/SF composites (**b**–**f**); PLA/SF/ADR composites (**A**); PLA/PBAT/SF/ADR composites (**B**–**F**). 40,000× magnification.

Figure 3 presents high magnification SEM micrographs of the phase morphology of PLA/PBAT composites on impact-fractured surfaces. It can be observed that the PBAT phase dispersed in the PLA continuous phase, and exhibited a typical “sea-island” phase structure. For PLA/SF and PLA/PBAT/SF composites (Figure 3a–f), the PBAT phase and PLA phase showed an obvious interface gap or debonding, indicating poor interfacial compatibility. However, for PLA/SF/ADR and PLA/PBAT/SF/ADR composites (Figure 3A–H), the size of the dispersed PBAT phase was minimized, and the interface between the PBAT phase and the PLA phase was well bonded. These phenomena revealed that the phase compatibility between PBAT and PLA was dramatically improved via the addition of ADR oligomer. Furthermore, elasto-plastic deformation of the PBAT phase could be observed as the PBAT content increased, especially in PLA/PBAT/SF/ADR composites with 15 wt% and 20 wt% PBAT content (Figure 3E,F). The addition of ADR oligomer enhanced the interfacial adhesion of sisal fibers and matrix as well as improved the interfacial compatibility of the PLA and PBAT phase. Al-Itry et al. proved the effect of ADR as an additive between PLA and PBAT in a paper on compatibilized PLA/PBAT blends [22]. As a result, the SEM images of PLA/PBAT/SF/ADR showed a feature of ductile fracture of matrix.

**Figure 4:**
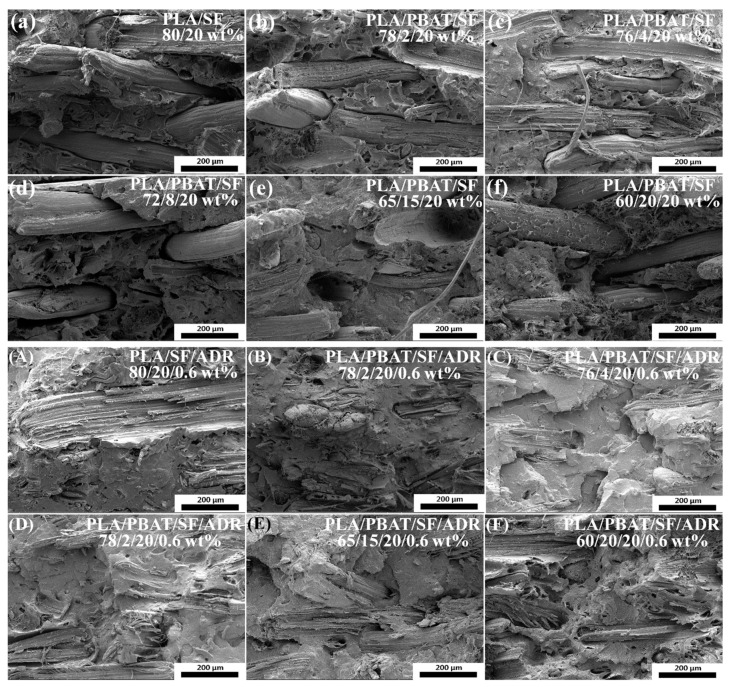
SEM micrographs of tensile fracture surfaces of PLA/SF composites (**a**); PLA/PBAT/SF composites (**b**–**f**); PLA/SF/ADR composites (**A**); PLA/PBAT/SF/ADR composites (**B**–**F**). 500× magnification.

When compared with the impact fracture, the tensile fracture constitutes a slow fracture-break process, and the debonding of fibers and matrix will be more significantly revealed in the composites. Figure 4 shows SEM micrographs of tensile fracture surfaces of composites. It can be seen from Figure 4a–f that the sisal fibers were directly pulled out from the matrix and the fibers almost retain the original state, which indicates the debonding of fibers from matrix during tensile tests, and the impairing of reinforcing effect of SF. However, the interfacial adhesion between SF and matrix in the composites with ADR oligomer was dramatically enhanced and it caused many fibers to be broken up or torn off in the tensile fracture (Figure 4A–F), which demonstrated that the sisal fibers in the composites bore loading in the tensile tests.

**Figure 5:**
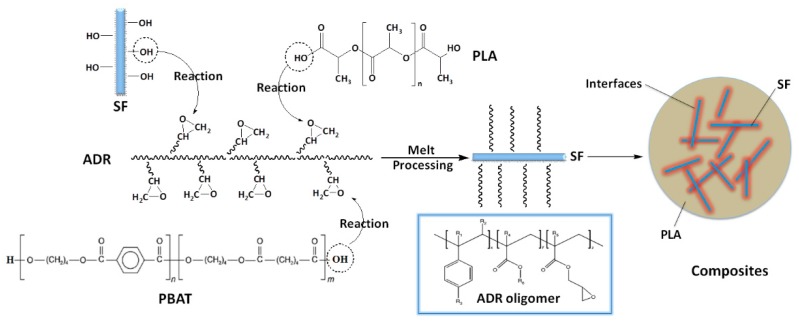
Illustration of interfacial compatibilization between PLA, SF, and PBAT via in-situ reaction with ADR oligomer during melt-blending processing.

In recent years, many modification methods of PLA resin have been based on the reaction ability of the end group of the PLA molecule chain, which are hydroxyl and carboxyl, by which typical works are reaction toughening of PLA [23,24,25,26,27]. For sisal fiber reinforced PLA composites, the presence of hydroxyl groups in the fiber surface has the potential to chemically bond with the PLA matrix. In this study, PLA, PBAT, and SF are in-situ reaction compatibilized via the addition of multi-epoxy-functionalized ADR oligomer during melt-blending processing. Figure 5 shows an illustration of interfacial compatibilization between PLA, SF, and PBAT via an in-situ reaction with the ADR oligomer during melt-blending processing. ADR oligomer might play a hinge-like role between sisal fibers and PLA or PBAT matrix, forming chemical bonds. SEM results demonstrated that ADR enhanced interfacial interaction between sisal fibers and matrix. The incorporation of ADR oligomer and PBAT resin has the potential to simultaneously reinforce and toughen PLA/SF composites.

### 3.2. FTIR Analysis of Extracted SF

**Figure 6:**
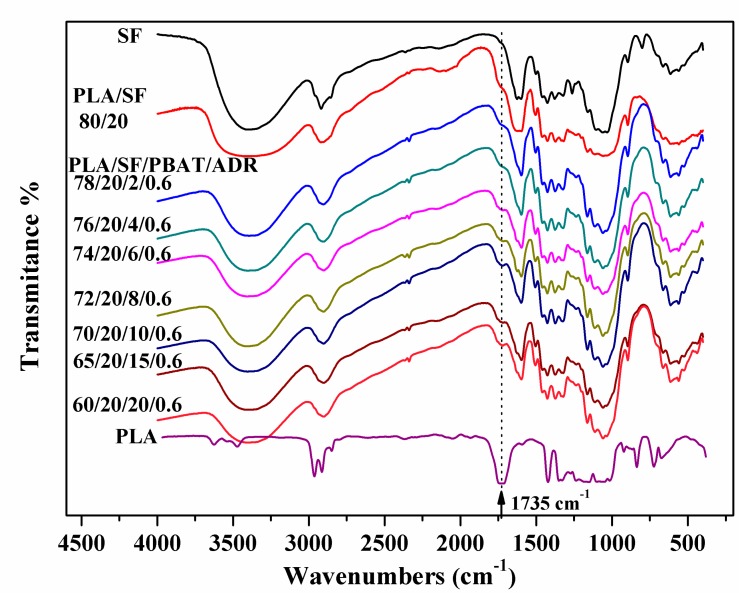
Fourier Transform infrared spectroscopy (FTIR) spectra of alkaline-treated SF, extracted SF from composites, and PLA resin.

Figure 6 shows the FTIR spectra of alkaline-treated SF, extracted SF from composites, and PLA resin to further prove the in-situ reaction of bonding matrix onto the fiber surface via melt-blending. The peak at 1735 cm^−1^ represents carbonyl (C=O) stretching vibration peak. This characteristic peak was not observed in alkaline-treated SF. However, it can be seen that the 1735 cm^−1^ peak presented in PLA/SF and PLA/SF/PBAT/ADR composites. For PLA/SF/PBAT/ADR composites, the intensity of this 1735 cm^−1^ peak increased with the addition of PBAT. It reflected that more addition of PBAT under existing ADR enhanced the chemical bonding of the PLA matrix to the sisal fibers during melt-blending processing. Therefore, more PLA bonded on the extracted SF from the composites. The result corresponded to the SEM analysis above.

### 3.3. Micro-Debonding Test Analysis

**Figure 7:**
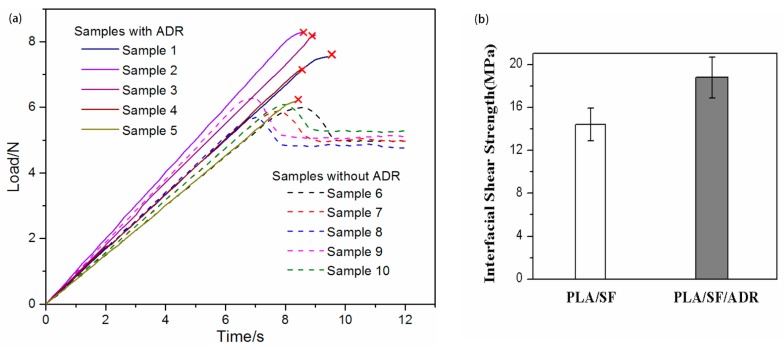
Curves of load vs time (**a**) and interfacial shear strengths (IFSS) (**b**) of micro-debond test samples with and without ADR.

In the process of micro-debonding test, both groups of samples showed different phenomena. For samples without ADR, the droplet debonded from the fiber when the tension reached to a point where the droplet began to slide along the fiber. Subsequently, the tension decreased and tended to be stable. For samples containing ADR, when the tension reached a certain level, the fiber fractured and no droplet debonding occurred, which indicated that the interfacial strength was beyond the strength of fiber itself. Figure 7 shows the curves of load vs time and the interfacial shear strengths (IFSS) calculated with maximum tension. The data indicated that IFSS of samples containing ADR was greater than that of samples without ADR. Moreover, the real IFSS of the sample containing ADR should be larger than the calculated value when the fiber broke. Therefore, it could be proved that the addition of ADR improved the interfacial strength between SF and PLA. The effect of the ADR on the interfacial shear strength between PLA and SF might be influenced by PBAT, as it can react with ADR during the melt blending process of the composites. However, ADR will certainly act as a compatibilizer between PLA and SF only if the mixing can be made properly to let ADR distribute uniformly inside the composite whereby it had a chance to simultaneously react with SF and PLA. Accordingly, it is reasonable to justify the interfacial shear strength of the composites with the presence of PBAT can be enhanced by the addition of ADR.

### 3.4. Mechanical Properties

**Figure 8:**
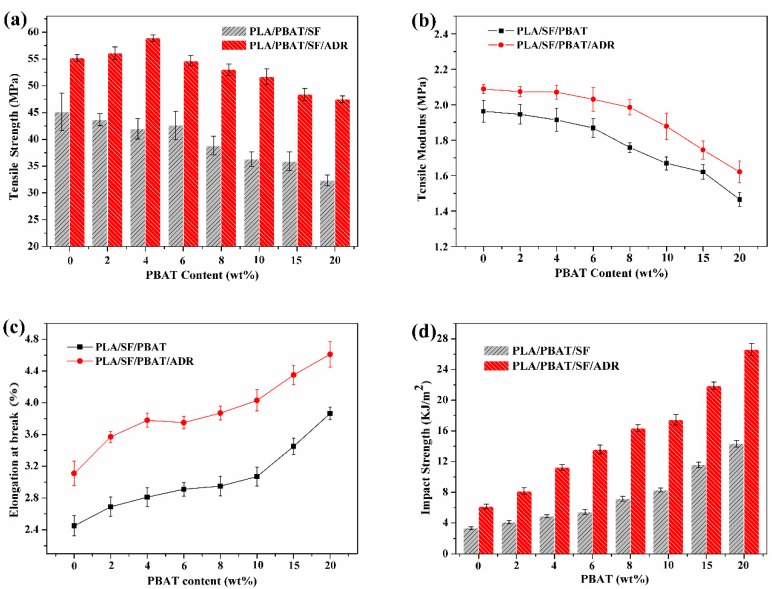
Tensile strength (**a**), tensile modulus (**b**), elongation at break (**c**), and impact strength (**d**) of PLA/PBAT/SF, and PLA/PBAT/SF/ADR composites.

Figure 8 shows the mechanical properties of PLA/PBAT/SF and PLA/PBAT/SF/ADR composites with different PBAT content. It indicated that the tensile strength, tensile modulus, elongation at break, and impact strength of PLA/PBAT/SF/ADR composites were improved in comparison with those of the PLA/PBAT/SF composites. That is to say, the stiffness and toughness of PLA/PBAT/SF/ADR composites were enhanced. Regarding the above-mentioned SEM morphology analysis, interfacial adhesion between SF and matrix was improved via the in-situ interfacial reaction with ADR oligomer. The improved interfacial interaction of PLA/PBAT/SF/ADR composites augmented the efficiency of transmitting stress from matrix to sisal fibers, thus improving the mechanical properties of the composites [28]. In addition, the interfacial compatibility between PBAT and PLA matrix of PLA/PBAT/SF/ADR composites was also enhanced, as shown in Figure 3, which contributed to the improvement of impact strength. For PLA/PBAT/SF composites, tensile strength decreased as the PBAT content increased. However, the tensile strength of PLA/PBAT/SF/ADR composites was improved in low PBAT content (2 and 4 wt%) and then decreased in high PBAT content. Moreover, it was found that the tensile strength of the PLA/PBAT/SF/ADR composites exceeded that of PLA/PBAT/SF composites, even at high PBAT content. Figure 8c,d also showed that the elongation at break and the impact strength of both kinds of composites ascended with increasing of PBAT content. The results further verified the above-mentioned morphology analysis that the implication of PBAT and ADR improved the plasticization effect of the mixture. Therefore, PLA/PBAT/SF/ADR composites exhibited strengthening and toughening mechanical properties, as compared with the PLA/SF composites.

**Figure 9:**
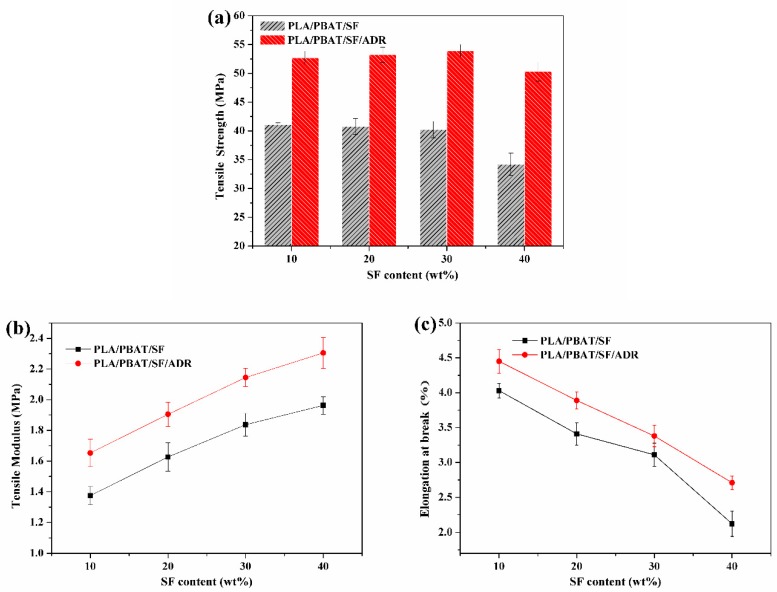
Tensile strength (**a**), tensile modulus (**b**), and elongation at break (**c**) of PLA/PBAT/SF and PLA/PBAT/SF/ADR composites.

**Figure 10:**
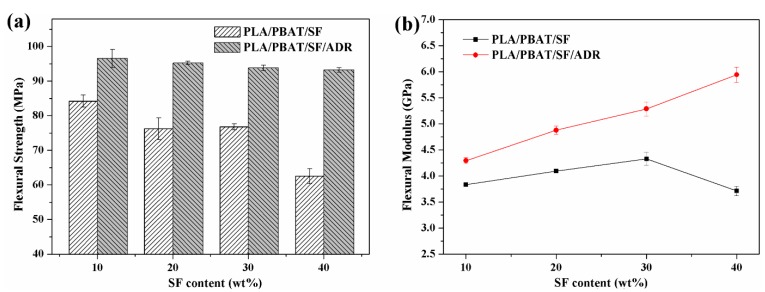
Flexural strength (**a**) and flexural modulus (**b**) of PLA/PBAT/SF and PLA/PBAT/SF/ADR composites with different SF content.

Figure 9 presents the tensile properties of PLA/PBAT/SF and PLA/PBAT/SF/ADR composites with different SF content and constant PBAT content of 8 wt%. With the increase of SF content, the tensile strength of PLA/PBAT/SF/ADR and PLA/PBAT/SF remained almost stable and declined to an extent at a high SF content of 40 wt%. However, the tensile strength of PLA/PBAT/SF/ADR was always elevated when compared with that of PLA/PBAT/SF. The tensile modulus of both kinds of composites continued to rise with SF content, but the change tendency of elongation at break reversed. The tensile strength, tensile modulus, and elongation at break of PLA/PBAT/SF/ADR composites were improved, as compared with those of PLA/PBAT/SF composites. In addition, the improvement was more distinct in high SF content. Figure 10 shows the flexural strength and flexural modulus of PLA/PBAT/SF and PLA/PBAT/SF/ADR composites with different SF content. It can be seen that, as SF content increased, the flexural strength of PLA/PBAT/SF composites decreased (Figure 10a), while the flexural modulus increased, except 40 wt% SF addition (Figure 10b). For plant fiber-reinforced polymer composites, the interfacial compatibility between plant fibers and matrix is generally poor due to the hydrophilic nature of plant fiber and the hydrophobic nature of polymer matrix. Even with just alkaline pretreatment, the mechanical properties of plant fiber-reinforced polymer matrix composites cannot reach an expected level and usually decrease at a high addition of plant fibers. The more plant fibers added, the weak interface between plant fiber and polymer matrix would be prominent. Nevertheless, the modulus of plant fibers-reinforced polymer composites commonly tended to increase with the addition of fibers, unless too many fibers were employed. However, Figure 10 showed that, when compared with PLA/PBAT/SF composites, the flexural strength of PLA/PBAT/SF/ADR composites decreased just a little and their flexural modulus obviously ascended with the increase of SF content. These phenomena demonstrated that ADR oligomer could promote the fiber reinforcing effect with enhanced interfacial bonding, even in high SF content.

The low interfacial bonding of PLA/SF composites caused composites to tend to easily debond under load. In addition, the poor interfacial adhesion of PLA/PBAT/SF composites resulted in microstructure defects of composites (Figure 1 and Figure 2) and stress concentration points when force was applied, which is undesirable in terms of mechanical properties of composites. Enhancing interfacial compatibility between fibers and matrix contributed to the formation of a moderate interface in composites, which can effectively relieve the stress concentration under loading conditions, and make the stress transmit uniformly from matrix to fibers [29,30]. For PLA/PBAT/SF composites with different SF content, more sisal fibers were added, more microstructure defects presented in composites, which incurred a decrease of mechanical properties of PLA/SF composites as the SF content increased. Attributed to the improved interfacial adhesion of composites induced by the addition of ADR oligomer, the mechanical properties of PLA/PBAT/SF/ADR composites were maintained at a relatively high level. Therefore, PLA/SF composite samples had the poorest mechanical properties, the toughness of PLA/PBAT/SF composites improved, and the PLA/PBAT/SF/ADR composites exhibited outstanding performance. Moreover, the elongation at the break of PLA/PBAT/SF/ADR composites was the largest in comparison with that of PLA/SF composites and PLA/PBAT/SF composites. 

## 4. Conclusions

In this study, polylactic acid/sisal fiber composites were fabricated while using an in-situ reactive melt-blending method with the application of epoxy-functionalized oligomer and poly (butylene-adipate-terephthalate). During melt blending and processing, ADR oligomer behaved like a hinge among sisal fibers and PLA/PBAT via an in-situ interfacial reaction. As a result, an improved interfacial interaction between fibers and matrix was achieved. Morphology characterization illustrated the enhanced interfacial interaction of PLA/PBAT/SF/ADR composites, and FTIR analysis confirmed the chemical bonding of matrix with SF in composites. In addition, the incorporation of PBAT exerted a plasticizing effect on composites, which softened the PLA matrix and augmented the toughness of PLA/SF composites. The micro-debonding test proved that the interfacial adhesion between PLA and SF was improved due to ADR. In comparison with PLA/SF and PLA/PBAT/SF composites, the mechanical properties of PLA/PBAT/SF/ADR composites were enhanced because of the improved interfacial compatibility between sisal fibers and polymer matrix. The simultaneous addition of PBAT and ADR facilitated composites to obtain a good stiffness-toughness balanced mechanical property, improving the toughness of PLA/SF composites without significant decline of tensile strength. 

## Figures and Tables

**Table 1:** Properties of sisal fibers.

Fiber Diameter (μm)	Fiber Density (g/cm^3^)	Cellulose Content (%)	Hemicellulose Content (%)	Lignin Content (%)
25–200	1.45	67–78	10–14	8–11

## References

[1] Mohanty A.K., Vivekanandhan S., Pin J.M., Misra M. (2018). Composites from renewable and sustainable resources: Challenges and innovations. Science.

[2] Tran L.Q.N., Fuentes C.A., Dupont-Gillain C., Van Vuure A.W., Verpoest I. (2013). Understanding the interfacial compatibility and adhesion of natural coir fibre thermoplastic composites. Compos. Sci. Technol..

[3] Thakur V.K. (2015). Interfacial Adhesion in Natural Fibre-Reinforced Polymer Composites. Lignocellulosic Polymer Composites: Processing, Characterization and Properties.

[4] Faruk O., Bledzki A.K., Fink H.P., Sain M. (2012). Biocomposites reinforced with natural fibers: 2000-2010. Prog. Polym. Sci..

[5] Kabir M.M., Wang H., Lau K.T., Cardona F. (2012). Chemical treatments on plant-based natural fibre reinforced polymer composites: an overview. Compos. Part B.

[6] Yu T., Ren J., Li S.M., Yuan H., Li Y. (2010). Effect of fiber surface-treatments on the properties of poly(lactic acid)/ramie composites. Compos. Part A.

[7] Xie Y.J., Hill C.A.S., Xiao Z.F., Militz H., Mai C. (2010). Silane coupling agents used for natural fiber/polymer composites: A review. Compos. Part A.

[8] Bledzki A.K., Mamun A.A., Lucka-Gabor M., Gutowski V.S. (2008). The effects of acetylation on properties of flax fibre and its polypropylene composites. Express Polym. Lett..

[9] Wang B., Panigrahi S., Tabil L., Crerar W. (2007). Pre-treatment of flax fibers for use in rotationally molded biocomposites. J. Reinf. Plast. Compos..

[10] Zahran M.K., Rehan M.F. (2006). Grafting of acrylic acid onto flax fibers using Mn(IV)-citric acid redox system. J. Appl. Polym. Sci..

[11] Kalia S., Kaith B.S., Kaur I. (2009). Pretreatments of natural fibers and their application as reinforcing material in polymer composites—a review. Polym. Eng. Sci..

[12] Zhou M., Li Y.H., He C., Jin T.X., Wang K., Fu Q. (2014). Interfacial crystallization enhanced interfacial interaction of Poly (butylene succinate)/ramie fiber biocomposites using dopamine as a modifier. Compos. Sci. Technol..

[13] Liu W.D., Xie T.S., Qiu R.H., Fan M.Z. (2015). N-methylol acrylamide grafting bamboo fibers and their composites. Compos. Sci. Technol..

[14] Sgriccia N., Hawley M.C., Misra M. (2008). Characterization of natural fiber surfaces and natural fiber composites. Compos. Part A.

[15] Farahani G.N., Ahmad I., Mosadeghzad Z. (2012). Effect of fiber content, fiber length and alkali treatment on properties of kenaf fiber/UPR composites based on recycled PET wastes. Polym. Plast. Technol. Eng..

[16] Thakur V.K. (2015). Chemical Modification and Properties of Cellulose-Based Polymer Composites. Lignocellulosic Polymer Composites: Processing, Characterization and Properties.

[17] Villalobos M., Awojulu A., Greeley T., Turco G., Deeter G. (2006). Oligomeric chain extenders for economic reprocessing and recycling of condensation plastics. Energy.

[18] Al-Itry R., Lamnawar K., Maazouz A., Billon N., Combeaud C. (2015). Effect of the simultaneous biaxial stretching on the structural and mechanical properties of PLA, PBAT and their blends at rubbery state. Eur. Polym. J..

[19] Arruda L.C., Magaton M., Bretas R.E.S., Ueki M.M. (2015). Influence of chain extender on mechanical, thermal and morphological properties of blown films of PLA/PBAT blends. Polym. Test..

[20] Li B., Zhang Y.H., Wu G.H. (2013). Thermoplastics reinforced by self-welded glass fibers: Effect of interfacial affinity on preferential segregation. Polymer.

[21] Xiu H., Qi X., Liu Z., Zhou Y., Bai H., Zhang Q., Fu Q. (2016). Simultaneously reinforcing and toughening of polylactide/carbon fibre composites via adding small amount of soft poly(ether)urethane. Compos. Sci. Technol..

[22] Al-Itry R., Lamnawar K., Maazouz A. (2014). Rheological, morphological, and interfacial properties of compatibilized PLA/PBAT blends. Rheol. Acta.

[23] Liu H.Z., Song W.J., Chen F., Guo L., Zhang J.W. (2011). Interaction of Microstructure and Interfacial Adhesion on Impact Performance of Polylactide (PLA) Ternary Blends. Macromolecules.

[24] Zhang K.Y., Nagarajan V., Misra M., Mohanty A.K. (2014). Supertoughened Renewable PLA Reactive Multiphase Blends System: Phase Morphology and Performance. ACS Appl. Mater. Interfaces.

[25] Ojijo V., Ray S.S. (2015). Super toughened biodegradable polylactide blends with non-linear copolymer interfacial architecture obtained via facile in-situ reactive compatibilization. Polymer.

[26] Wu M., Wu Z.Q., Wang K., Zhang Q., Fu Q. (2014). Simultaneous the thermodynamics favorable compatibility and morphology to achieve excellent comprehensive mechanics in PLA/OBC blend. Polymer.

[27] Hao M., Wu H.W., Zhu Z. (2017). In situ reactive interfacial compatibilization of polylactide/sisal fiber biocomposites via melt-blending with an epoxy-functionalized terpolymer elastomer. RSC Adv..

[28] Ma L.C., Meng L.H., Wu G.S., Wang Y.W., Zhao M., Zhang C.H., Huang Y.D. (2015). Improving the interfacial properties of carbon fiber-reinforced epoxy composites by grafting of branched polyethyleneimine on carbon fiber surface in supercritical methanol. Compos. Sci. Technol..

[29] Jiang D.W., Xing L.X., Liu L., Yan X.R., Guo J., Zhang X., Zhang Q.B., Wu Z.J., Zhao F., Huang Y.D. (2014). Interfacially reinforced unsaturated polyester composites by chemically grafting different functional POSS onto carbon fibers. J. Mater. Chem. A.

[30] Jiang D.W., Liu L., Long J., Xing L.X., Huang Y.D., Wu Z.J., Yan X.R., Guo Z.H. (2014). Reinforced unsaturated polyester composites by chemically grafting amino-POSS onto carbon fibers with active double spiral structural spiralphosphodicholor. Comp. Sci. Technol..

